# Case report: Dezocine’s rapid and sustained antidepressant effects

**DOI:** 10.3389/fphar.2024.1411119

**Published:** 2024-07-18

**Authors:** Han Wang, Nan Lyu, Qian Zhao

**Affiliations:** ^1^ Beijing Key Laboratory of Mental Disorders, National Clinical Research Center for Mental Disorders and National Center for Mental Disorders, Beijing Anding Hospital, Capital Medical University, Beijing, China; ^2^ Laboratory for Clinical Medicine, Capital Medical University, Beijing, China; ^3^ The Advanced Innovation Center for Human Brain Protection, Capital Medical University, Beijing, China

**Keywords:** major depressive disorder, opioid, dezocine, anhedonia, case

## Abstract

Anhedonia and motivational impairments are cardinal features of depression, against which conventional antidepressants demonstrate limited efficacy. Preclinical investigations and extant clinical trial data substantiate the promise of opioid receptor modulators in addressing anhedonia, depression, and anxiety. While synthetic opioid agents like dezocine are conventionally employed for analgesia, their distinctive pharmacological profile has engendered interest in their potential antidepressant properties and translational applications. Herein, we present a case in which persistent bupropion treatment was ineffective. However, the incidental administration of a single low-dose intravenous injection of dezocine resulted in a rapid and sustained amelioration of depressive symptoms, particularly anhedonia and motivational deficits. Our findings posit a potentially novel role for the “legacy drug” dezocine.

## Introduction

Major depressive disorder (MDD) is a frequently encountered mood disorder characterized by a high prevalence, diagnostic challenges, and significant disability rates. Anhedonia, a core symptom of MDD, serves as a poor prognostic indicator of antidepressant response (Klein et al., 2022). Research indicates that anhedonia affects 37%–72% of individuals with MDD, contributing to heightened morbidity and increased suicidality (Tang et al., 2021). Although monoaminergic antidepressants are the standard first-line treatment for MDD, their effectiveness in alleviating anhedonia remains limited (Shawn et al., 2011). Currently, no medication has been approved for the treatment of anhedonia. This highlights the need for effective and expedited interventions that specifically target this symptom.

Emerging evidence has elucidated the pivotal involvement of endogenous opioid receptors, including μ-opioid receptor (MOR), κ-opioid receptor (KOR), δ-opioid receptor (DOR), and nociceptin/orphanin FQ peptide receptor, in the pathophysiology of MDD (Puryear et al., 2020). These receptors are widely distributed in regions associated with emotional processing and reward systems, notably the mesocorticolimbic system, and play a significant role in modulating pleasurable experiences (Puryear et al., 2020). Imaging modalities have revealed diminished activity in the endogenous opioid system among individuals with MDD, which is correlated with impaired emotional regulation and decreased pleasurable experiences (Nummenmaa et al., 2020). Notably, the evidence from several clinical investigations has substantiated that the administration of multimodal opioid agonists, such as ALKS–5461 (Fava et al., 2020), JNJ–67953964 (Krystal et al., 2020), and BTRX–246040 (Post et al., 2016), either as monotherapy or in conjunction with other treatments, is associated with substantial antidepressant efficacy and a beneficial effect on the mitigation of anhedonia. Preclinical investigations have shown that MOR activation enhances rewarding experiences, whereas KOR activation is generally linked to diminished pleasure. DOR activation indirectly promotes dopamine release from the nucleus accumbens, thereby regulating reward-related behaviors (Browne, 2019). Strong clinical and preclinical evidence supports the potential of opioid-based pharmacotherapy in the management of MDD, particularly for the amelioration of anhedonic manifestations.

**FIGURE 1:**
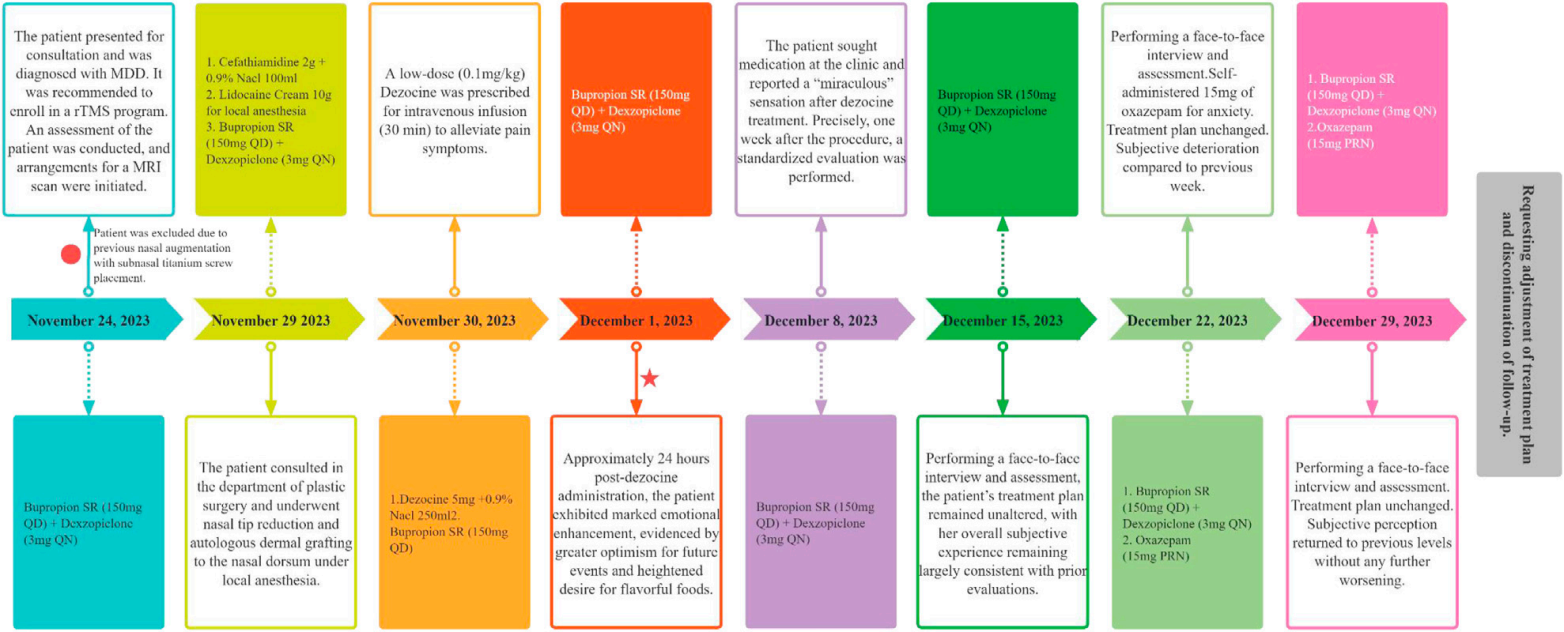
Timeline of event onset and evaluation of symptomatic presentation with an outline of therapeutic intervention.

Dezocine, a synthetic opioid with structural similarities to benzomorphan opioids, is primarily used for the management of moderate-to-severe pain in clinical settings (Ye et al., 2022). Functionally, it acts as a partial agonist/antagonist at MOR, demonstrates partial agonism at DOR, and modulates KOR to varying degrees (Liu et al., 2014; Wang et al., 2018). Additionally, dezocine inhibits the norepinephrine transporter (NET) and serotonin transporter (SERT), thereby impeding the reuptake of the respective neurotransmitters (Liu et al., 2014). The clinical efficacy of dezocine is comparable to that of morphine and other opioids, while displaying a lower potential for addiction and a reduced incidence of side effects (Wang et al., 2017). Despite its established analgesic use, limited research has been conducted on the antidepressant properties of dezocine, despite its interaction with key targets such as MOR, KOR, NET, and SERT. Initial preclinical research indicates that dezocine may alleviate depression-like behavior in rodents in a dose-dependent manner (Shang et al., 2021) and has shown promise in the clinical attenuation of postoperative depression following cancer resection (Zhao et al., 2020). Despite the limited evidence regarding the antidepressant properties of dezocine, its distinct pharmacodynamic profile necessitates a comprehensive investigation into its potential efficacy and mechanism of action in the treatment of depression. Herein, we present a recent case of MDD in which rapid and sustained improvement in depressive mood and anhedonia was observed following dezocine administration (Figure 1).

## The case report

A young woman who initially presented with MDD in June 2014 was diagnosed therewith at multiple psychiatric hospitals in China. She was previously prescribed various antidepressants, including escitalopram, paroxetine, and venlafaxine. Treatment adherence was satisfactory, with moderate efficacy observed. The predominant rationale for altering pharmacotherapy was an unwillingness to tolerate medication-induced weight gain. Prior to this depressive episode, she had experienced a remission period of up to 2 years. However, following her mother’s death a year ago, the patient experienced a recurrence of symptoms. The outpatient physician initially prescribed venlafaxine, which had previously been effective for the patient. However, with ongoing treatment, the patient’s anhedonia symptoms worsened. Consequently, the treatment regimen was gradually switched to bupropion. And so far, she was prescribed sustained-release bupropion (150 mg) for nearly 3 months.

The patient was in good physical condition. Underwent rhinoplasty in April 2022 with titanium screw placement at the nasion. Primogeniture among three siblings with normative prenatal exposure and developmental progression. The individual had attained an undergraduate education and was engaged in a profession within the social media sector. There was no history of matrimony or parenthood. Notably, there existed a positive familial history, with a diagnosis of a mood disorder in a sibling, confirmed by a specialized psychiatric facility.

On 24 November 2023, the patient was enrolled in a clinical trial involving repetitive transcranial magnetic stimulation (rTMS) and was diagnosed with MDD using the Mini-International Neuropsychiatric Interview. During the baseline phase, the participants’ symptoms were quantitatively assessed using standard psychometric scales, including the Hamilton Depression Rating Scale (HAMD-17), Montgomery–Asberg Depression Rating Scale, Patient Health Questionnaire-9, and Dimensional Anhedonia Rating Scale (DARS). The results indicated moderate depressive symptoms and severe anhedonia (Table 1). Prior to the MRI scan, the subject recalled having undergone nasal augmentation with subnasal titanium screw placement. Given the potential risks associated with metal objects and magnetic fields in rTMS, the investigative team recommended extraction of the titanium nasal screw prior to the initiation of the study protocol to ensure patient safety.

**TABLE 1:** Quantitative assessment results of the Patient’s depressive symptoms and anhedonia.

	Baseline	1 week*	2 weeks*	3 weeks*	4 weeks*
Depressive assessment					
HAMD-17	18	2	5	10	17
MADRS	23	4	9	15	22
PHQ-9	14	1	2	8	12
Anhedonic assessment					
DARS (Total)	17	52	49	35	28
Hobbies	3	15	15	9	9
Food and drink	4	9	5	8	7
Social activities	4	10	10	4	1
Sensory experience	6	18	19	14	11
Item 7 of HAMD-17	4	0	0	2	3
Item 8 of MADRS	4	0	0	2	4
Item 1 of PHQ-9	3	0	0	1	3

* Represents the time after dezocine was administered intravenously.

Abbreviations: HAMD-17, Hamilton Depression Rating Scale-17; MADRS, Montgomery-Asberg Depression Rating Scale; PHQ-9, Patient Health Questionnaire-9; DARS, Dimensional Anhedonia Rating Scale.

From the November 29 to 1 December 2023, the patient underwent elective removal of a titanium nasal screw and concurrent rhinoplasty (specifically, nasal tip refinement) in the plastic surgery department. Postoperatively, on 30 November 2023, the patient was prescribed an analgesic regimen consisting of a low-dose intravenous dezocine injection at 0.1 mg/kg. The prescribed dezocine (5 mg in total) was diluted in 250 mL of 0.9% sodium chloride solution and administered via infusion over 30 min. Apart from the reported tolerable nausea, no additional adverse effects were noted by the patient and caregivers.

Approximately 24 h after dezocine administration, the patient reported a remarkable and spontaneous enhancement in emotional wellbeing, characterized by a reinvigorated zest for life and cravings for gourmet food. The patient’s subjective experience was described as a transition from feeling emotionally “empty” to a sensation of embodying their authentic self. Regrettably, no formal quantitative evaluations were conducted. During the trial, the patient consistently received 150 mg of sustained-release bupropion and 3 mg of dexzopiclone. One week after the initiation of dezocine treatment, the patient returned to the hospital to receive antidepressants and declined further participation in the rTMS program. The patient recounted her “miraculous” experience, prompting an immediate comprehensive evaluation and subsequent weekly face-to-face follow-ups. The results demonstrated a significant amelioration of depressive symptoms and anhedonia by the end of the first and second weeks, with 88.89% and 72.22% reductions in the HAMD-17 scores, respectively, as outlined in Table 1. Additionally, there was a notable improvement in the patient’s anhedonia, with weekly increase in DARS scores by 35 and 32 points above the baseline, respectively. By the third week, the patient’s reduction rate on HAMD-17 subsided to 44.44%, and the DARS scores decreased to 35 points. At the end of the fourth week, the patient’s symptoms had exacerbated, described as “almost as before,” and assessments were essentially congruent with pre-dezocine treatment baselines, prompting the patient to express a desire for augmented therapeutic intervention. Consequently, follow-up was discontinued, and the treatment plan was amended. During prolonged follow-up, the patient notably expressed appreciation for recent experiences via social media, without indicating any adverse progression or augmented dependency post-dezocine administration, advocating for additional clinical research.

## Discussion

To our knowledge, this is the first clinical report of dezocine ameliorating depressive experiences and anhedonic symptoms in MDD, characterized by both a rapid onset of action and sustained effects, without evidence of rebound phenomena. This can be attributed to the distinct pharmacological properties of dezocine. Given the clinical observation that the efficacy of serotonin-norepinephrine reuptake inhibitors, blocking the activity of NET and SERT, is often delayed, the rapid antidepressant effect of dezocine may be associated with opioidergic modulation rather than monoaminergic systems alone. Synergistic potentiation between the opioidergic and monoaminergic systems cannot be excluded, especially given the evidence that ketamine can expedite the antidepressant onset of escitalopram and boost its efficacy (Xiao et al., 2023). The sustained antidepressant effects of dezocine, with an average terminal half-life of approximately 2.4 h, raise questions as to whether this reflects placebo effects, reactivation of antidepressants, slow metabolism subsequent to receptor occupancy similar to ketamine (Ma et al., 2023), warranting further investigation.

Furthermore, it is noteworthy that the patient’s background antidepressant is sustained-release bupropion. Given the recent FDA approval of the novel antidepressant dextromethorphan HBr-bupropion HCl (Auvelity) (Tabuteau et al., 2022), it is conceivable that the sustained therapeutic effect of dezocine might be attributed to prolonged exposure by competitive inhibition of cytochrome P450 2D6 (CYP 2D6) by bupropion, especially since studies have documented the impact of the CYP 2D6 genotype on the pharmacodynamics and pharmacokinetics of opioid medications (Ballester et al., 2022).

Addiction is the most politically charged adverse effect of opioid pharmacotherapy. However, the extant evidence for its addictive liability remains scant. In animal models, dezocine has even been observed to antagonize addiction-like behaviors induced by opioids such as morphine (Wu et al., 2019). Although it has the low potential to induce tolerance and dependence compared to more potent opioids, caution is warranted considering the protracted nature of antidepressant therapy. Alternatively, future research might actively investigate derivatives that circumvent the risk of addiction, along with rational dosing regimens.

While the current evidence is limited to case reports, the pharmacological profile of dezocine suggests that it has significant potential for the treatment of MDD, particularly in patients manifesting anhedonic symptoms. This has been indirectly validated in studies on buprenorphine, a compound with similar mixed opioid receptor activity (Namchuk et al., 2022). Compared to buprenorphine, dezocine’s blockade of SERT and NET appears to more robustly support its antidepressant effects. However, the role of dezocine on KOR remains uncertain, with its agonist or antagonist activity at different doses yet to be clarified (Ye et al., 2022). This highlights the need for high-quality clinical and preclinical research to corroborate these findings and develop viable therapeutic protocols. Moreover, the opioidergic system is necessary but insufficient component for the antidepressant effects of ketamine (Klein et al., 2020). In conjunction with this case report, dezocine exhibited rapid and sustained antidepressant clinical effects, similar to those of ketamine (Grunebaum et al., 2018). However, dezocine is devoid of dissociative manifestations, whether dezocine possesses the potential to emerge as next ketamine in clinical applications still requires validation through large-scale, high-quality clinical studies.

## Data Availability

The original contributions presented in the study are included in the article/Supplementary Material, further inquiries can be directed to the corresponding author.

## Abbreviations

MDD, major depressive disorder; rTMS, repetitive transcranial magnetic stimulation; QD, quaque die; QN, quaque night; PRN, pro re nata.
